# Proposal of a diagnostic algorithm for radiation-induced dropped head syndrome in long-term childhood cancer survivors based on a prospective study in a specialized clinical setting and a review of the literature

**DOI:** 10.1007/s00432-023-05480-w

**Published:** 2023-11-10

**Authors:** Sarah Rieken, Lea Louisa Kronziel, Thorsten Langer, Dirk Rades, Tobias Boppel, Peter Trillenberg, Judith Gebauer

**Affiliations:** 1 Department of Oncology and Hematology, University Medical Center Schleswig-Holstein, Ratzeburger Allee 160, 23538 Luebeck, Germany; 2 Institute of Medical Biometry and Statistics, University of Luebeck, Luebeck, Germany; 3 Department of Pediatric Oncology and Hematology, University Medical Center Schleswig-Holstein, Ratzeburger Allee 160, 23538 Luebeck, Germany; 4 Department of Radiation Oncology, University Medical Center Schleswig-Holstein, Ratzeburger Allee 160, 23538 Luebeck, Germany; 5 Department of Neuroradiology, University Medical Center Schleswig-Holstein, Ratzeburger Allee 160, 23538 Luebeck, Germany; 6 Department of Neurology, University Medical Center Schleswig-Holstein, Ratzeburger Allee 160, 23538 Luebeck, Germany; 7 Department of Internal Medicine I, University Medical Center Schleswig-Holstein, Ratzeburger Allee 160, 23538 Luebeck, Germany

**Keywords:** Dropped head syndrome, Long-term follow-up, Childhood cancer survivors, Late effects, Radiotherapy

## Abstract

**Purpose:**

To prospectively assess the incidence of Dropped Head Syndrome (DHS) in childhood cancer survivors (CCS) and to develop and evaluate a diagnostic algorithm for DHS.

**Methods:**

A systematic literature search for DHS in combination with neck radiotherapy (RT) exposure was performed. Analyses and a combination of the most common examination methods were integrated into a diagnostic algorithm. Almost all CCSs visiting the local late effects clinic between May 2020 and April 2022 were included in the study. CCS exposed to neck RT with doses ≥ 19 Gy received standardized clinical and neurological assessment and, in case of abnormal results, an MRI scan to confirm muscle atrophy.

**Results:**

Two hundred and five CCS were included of whom 41 received RT to the neck with ≥ 19 Gy. In the entire cohort and in the subgroup receiving RT, 2.4% and 12% of CCS were affected by DHS, respectively. Results of clinical and neurological assessment correlated well with MRI results. Neck circumference and neck/thigh ratio were lower after neck RT. Over 50% of CCS experienced neck disability and pain.

**Conclusions:**

A relevant proportion of CCS exposed to neck RT is affected by DHS. High concordance of MRI results with the neurological examination supports the clinical value of the diagnostic algorithm. Measurement of neck circumference might be an easy tool for assessment of neck muscle atrophy in survivors at risk.

**Implications for cancer survivors:**

Integration of a diagnostic algorithm for DHS in standard long-term follow-up care facilitates diagnosis as well as initiation of early treatment and obviates the need for invasive examinations.

**Supplementary Information:**

The online version contains supplementary material available at 10.1007/s00432-023-05480-w.

## Introduction

During the last decades, cancer survival rates have improved considerably for most entities resulting in a growing number of cancer survivors worldwide (Siegel et al. 2022; Kaatsch et al. 2022). Whereas in the beginning, the aim of cancer therapy was primarily to optimize the overall survival, in recent years, the focus has shifted more and more to living beyond cancer. Over two-thirds of childhood cancer survivors (CCS) develop chronic health conditions later in life that are often recognized as consequences of the former cancer treatment and affect survivors’ quality of life and long-term survival (Oeffinger et al. 2006; Bhakta et al. 2017).

In the beginning of standardized treatment protocols high dose extended field irradiation was implemented in the first Hodgkin’s Lymphoma’s (HL) treatment regimen (HD-78) (Bramswig et al. 1990). As excellent survival rates exceeding 90% resulted in a growing number of HL survivors, late effects of intensive cancer treatment could be observed in this group comparably early. Consequently, adaptations of treatment protocols were necessary to find the balance between cure and long-term toxicity (Bramswig et al. 1990; Calaminus et al. 2014). As late effects of cancer treatment usually occur after decades and depend on the intensity of treatment exposure, patients with long-term survival are especially at risk. Consequently, specialized long-term follow-up (LTFU) including regular screening examinations for known late effects is recommended to facilitate early detection and prevention of severe sequelae (Gebauer et al. 2020).

In Germany, multidisciplinary LTFU care for patients surviving > = 5 years after childhood cancer treatment is offered at several University Hospitals. Based on CCS’ treatment and personal risk factors, patients visit the clinic every one to five years and receive risk-adapted examinations (Gebauer et al. 2020). Risk-adapted LTFU screening recommendations are often based on analyses of survivors’ health status years to decades after treatment exposure in relation to the expected incidence of various diseases in the general population. However, rare sequelae might be missed with this approach. One of these rare late effects is weakness (and atrophy) of the muscles within the former radiation field which has been described as a dropped head syndrome (DHS) after neck radiotherapy (RT) as it might result in consecutive difficulty to hold the head up straight (van Leeuwen-Segarceanu et al. 2012; Rowin et al. 2006). Previous studies demonstrated that a considerable number of HL survivors report muscle weakness as well as pain and stiffness after being treated with mantle field radiation (van Leeuwen-Segarceanu et al. 2012). Although DHS represents a condition that occurs more commonly in elderly patients in the general population, there are case reports since the 1980s that describe DHS in younger patients after being treated with neck RT (Furby et al. 2010; Lange et al. 1986). Pathomechanisms of DHS are not entirely understood. It is predominantly attributed to fibrosis, contraction of the anterior cervical muscles, and atrophy of the posterior neck and shoulder girdle resulting in decreased neck muscle strength (Rowin et al. 2006). The risk of developing DHS increases with radiation doses to the neck and at the same time the latency seems to shorten after receiving higher radiation doses so that the onset varies from only a few years after radiotherapy up to > 20 years (Luo 2008; Inaba et al. 2016). Further risk factors associated with the manifestation of DHS are neck surgery (Luo 2008) and acceleration trauma (Schlienger et al. 2013). Additionally, chronic fatigue has been discussed as a common feature in survivors with decreased neck muscle strength (van Leeuwen-Segarceanu et al. 2012). Although single studies reported high incidences of neck muscle weakness in a selected, small cohort of long-term HL survivors treated with mantle field RT, occurring in up to 85% of patients (10 out of 12), no standard diagnostic approach for survivors at risk has been defined yet (van Leeuwen-Segarceanu et al. 2012). Moreover, in several cases reported so far, invasive diagnostic procedures potentially associated with complications of the examination, have been used, mainly to exclude other underlying diseases that can also manifest as DHS (Appels and Goekoop 2009). As a diagnosis of DHS is based predominantly on clinical and neurological characteristics, a better understanding of radiation-induced DHS and its associated risk factors may result in less but focused examinations. Furthermore, it remains unclear how many long-term survivors in an unselected cohort of CCS are affected by this sequela and if upfront screening and treatment could attenuate the natural course of this disease. Up until now, apart from a general reference in terms of RT-induced fibrosis in the Children’s Oncology Group (COG) survivor guideline, this topic has not been addressed in current LTFU recommendations (Hudson et al. 2021).

Consequently, in this study, our aim was to establish a reasonable diagnostic screening pathway for radiation-induced DHS, based on a review of the literature in a three-step selective process, and to evaluate this tool prospectively in an unselected cohort of CCS at our local late effects clinic.

## Participants and methods

### Systematic review

**Fig. 1:**
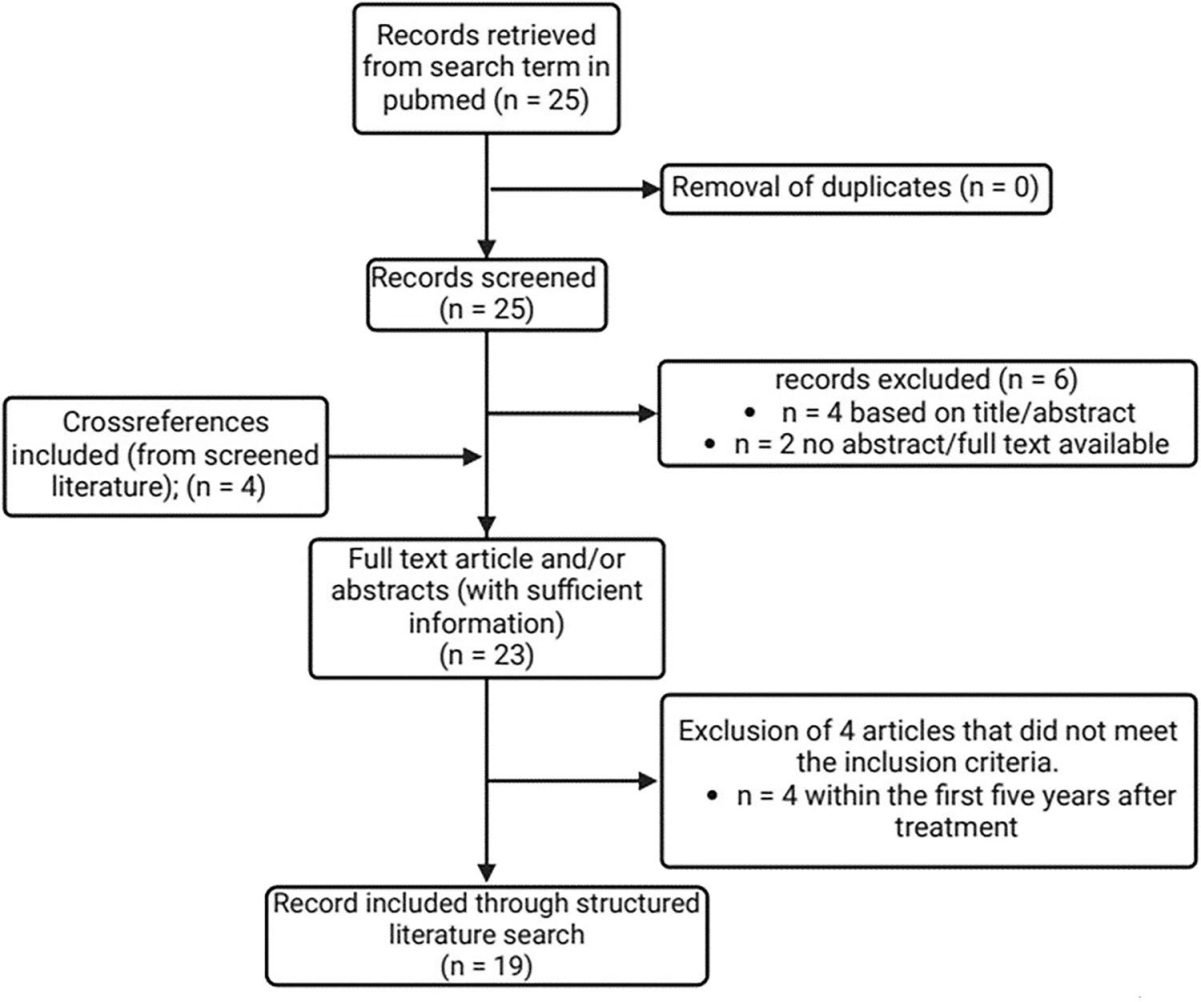
Illustration of the literature review process

We conducted a systematic literature search in the Pubmed database using the terms “dropped head syndrome” in combination with “cancer” or “radiotherapy”. We limited our search to articles in English; however, due to the small sample size, we included articles in other languages if an English abstract was available that allowed a clear assignment of the article’s content to DHS. Since the naming of the disease as DHS was inconsistent in the literature available, we also included cross-references if the condition described could be clearly identified as DHS after neck RT (e.g. progressive muscle atrophy and weakness; van Leeuwen-Segarceanu et al. 2012). As we focused on DHS as a late complication of RT, we only included articles presenting DHS 5 or more years after RT exposure (Fig. 1).

**Fig. 2:**
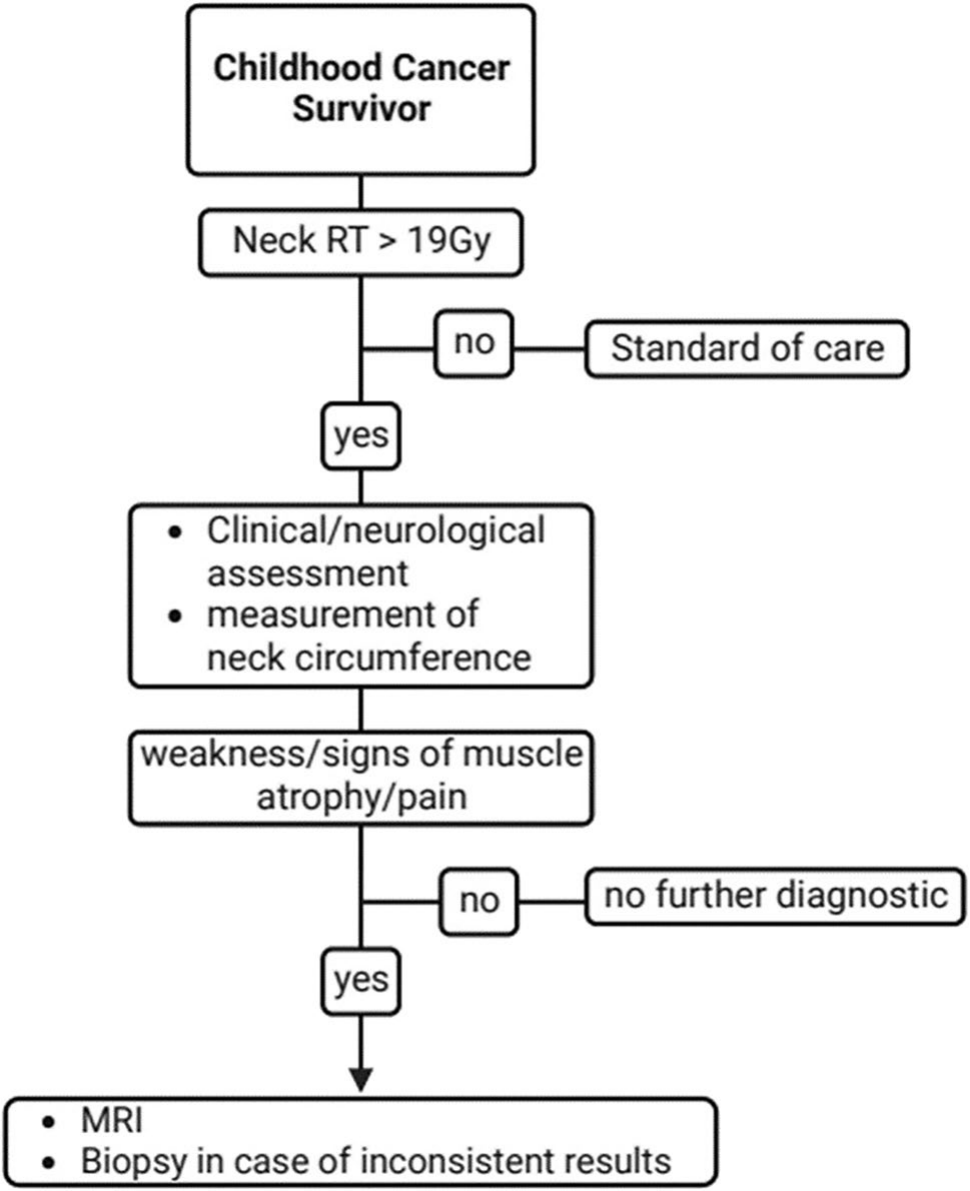
Proposed diagnostic algorithm for evaluation of radiation-induced DHS

The following diagnostic tools have been applied for diagnosis of radiation-induced DHS in the included articles (frequency in brackets): neurophysiological examination (16 out of 19), electromyogram (EMG) (11 out of 19), magnetic resonance imaging (MRI) (12 out of 19), biopsy (6 out of 19), computed tomography (CT) (6 out of 19) (Table 1). Based on this, we developed a diagnostic algorithm for radiation-induced DHS that was prospectively evaluated in our specialized late effects clinic (Fig. 2). MRI was preferred over CT due to radiation protection reasons. EMG has not been included in the diagnostic algorithm as former studies revealed conflicting results with both myopathic and/or neurogenic EMG alterations (Furby et al. 2010). Muscle biopsy showed primarily non-specific abnormalities in histopathological studies; however, as one study indicated the possibility of a specific underlying pathologic substrate such as nemaline myopathy that has been described as radiation-related disorders, histologic evaluation has been considered as “ultima ratio” for unsolved cases (Portlock et al. 2003).

**Table 1:** Review of the literature

No.	Author	Year	Type	Cases of post-radiotherapy DHS	Cancer	Delay after radiotherapy/time since diagnosis*	Gy	Irradiation field	Chemo-therapy	Diagnostics						
										MRI	CT	EMG	Physical/neurological examination	Muscle biopsy	Laboratory	Other diagnostic features
1	Appels and Goekoop (2009)	2009	Case report	2	HL	14 years + 31 years	NR	MF	NR	x	–	–	x	x	x	
2	Behin et al. (2008)	2008	Literature research	Not specified	Various, Mostly HL	10 to > 20 years		MF	NR	NR	NR	NR	x	Various	NR	
3	Brodell et al. (2020)	2020	Literature research	12 + 6	HL	5–30 years	NR	NR	NR	x	NR	NR	x	–	NR	
4	Caruso et al. (2014)	2014	Case report	1	HL	10 years	NR	MF	NR	–	–	–	x	–	–	Cervical radiograph
5	Di Stefano et al. (2018)	2018	Case report	1	HL	28 years	NR	MF	x	x	x	x	x	–	x	Nerve conduction, PET scan
6	Ellrichmann et al. (2016)	2016	Case report	1	HL	28 years	44 Gy	MF + Mediastinum	x	x	x	x	x	x	NR	Genetic testing, CSF analysis
7	Furby et al. (2010)	2009	Case report	6	HL	5–30 years	NR	MF	x	x	x	x	x	–	x	Nerve conduction, whole body PET, CSF analysis
8	Ghosh and Milone (2015)	2014	Case report	9	Various	2–45 years	35–75 Gy	Various	Various	Various	–	x	x	Various	x	
9	Grimm and Chamberlain (2011)	2011	Literature research	Not specified	HL	Many years	NR	MF	NR	NR	NR	x	x	Various	NR	
10	Hashimoto et al. (2012)	2012	Case report	1	NPC	2 years	64.8 Gy	Cervical irradiation	x	–	x	x	x	–	x	
11	Johansson et al. (1998)	1998	Questionnaire based survey	20	HL	NR	NR	NR	NR	–	–	–	–	–	–	
12	Kaido et al. (2018)	2018	Case report	1	HL	10 years	NR	NR	NR	x	x	x	x	–	x	
13	Kim and Stubblefield (2019)	2019	Questionnaire based survey	26	HL		NR	NR	NR	–	–	–	–	–	–	Questionnaire: prescription of a headmaster collar
14	Ochi et al. (2016)	2016	Case report	1	NSCLC	26 years	NR	MF	NR	NR	x	NR	NR	NR	NR	
15	Portlock et al. (2003)	2003	Case report	1	HL	16 years	35 Gy	MF + paraaortic lymph nodes	x	x	–	–	x	x	x	Nerve conduction studies
16	Rowin et al. (2006)	2006	Case report	3	HL	2–27 years	NR	MF, TBI	Various	x	–	x	x	–	x	Nerve conduction studies, cervical radiograph
17	Seidel et al. (2015)	2015	Case report + literature review	1 (+ 45)	NHL	23 years	45 Gy	Abdominal lymph nodes	x	x	–	x	x	–	x	
18	Schlienger et al. (2013)	2012	Case report	1	HL	27 years	40 Gy	MF	x	x	–	x	x	–	–	
19	Witvoet and Wirtz (2019)	2019	Case report	1	HL	30 years	NR	MF	NR	x	–	x	x	–	x	

*CSF* cerebrospinal fluid, *HL* Hodgkin’s lymphoma, *m* months *MF* mantle field irradiation, *NHL* non-Hodgkin lymphoma, *NPC* nasopharyngeal carcinoma, *NSCLC* non-small-cell lung cancer, *NPC* nasopharyngeal carcinoma, *NR* not reported data, *TBI* total body irradiation

*Delay after radiotherapy may not be specified, but timespan since cancer diagnosis may be given

### Study population

Almost all CCS visiting our local late effects clinic between May 2020 and April 2022 have been included in this study. Only one patient withdrew his consent, all others participated and will be referred to as CCS in the following. The LTFU clinic at the university hospital Schleswig Holstein, Campus Luebeck, was established in 2014 and currently treats around 200 CCS per year (Gebauer et al. 2018). Survivors who were 18 years or older at the time of cancer diagnosis but were formerly treated in the pediatric oncology department according to pediatric oncology treatment protocols (e.g. medulloblastoma patients) and regularly visit our local late effects clinic have been included in this study. Details regarding cancer treatment including chemotherapeutic agents as well as dose and target volume parameters of RT were obtained from former medical reports.

The recruitment period was prolonged from 12 to 23 months due to the cessation of the outpatient clinic during the COVID-19 pandemic to include all CCS with exposure to neck RT. These CCS are generally advised to visit the late effects clinic once per year, based on the risk stratification published in 2020 (Gebauer et al. 2020). In this summary of international LTFU recommendations, neurological examination following neck RT is not regularly recommended.

### Clinical examination

All participants at the late effects clinic received measurements of thigh, waist and neck circumference. Neck circumference (cm) was measured using non-stretchable plastic tape to the nearest 1 mm and was measured from the level just below the laryngeal prominence perpendicular to the long axis of the neck with the head positioned in the Frankfurt horizontal plane (according to Anothaisintawee et al. 2019). Cancer- and treatment-related data have been extracted from the local LTFU database. Chronic diseases with a possible effect on neuromuscular function (such as untreated hypothyroidism) have been excluded (Burakgazi et al. 2019).

### Neurological examination

All CCS who received at least 19 Gy of neck RT were defined as the exposed group and received a structured neurological examination. The cutoff for radiation exposure was set at 19 Gy based on standard treatment protocols for HL, which allowed us to include all HL survivors exposed to neck RT. Examinations included reflexes testing, clinical evaluation of muscle strength in the arms according to the MRC scale as well as the ability to perform three Hammersmith Functional Motor Scale (HFMS) exercises. Muscle strength was also measured with a hand-held dynamometer (Citec dynamometer CT 3001, C.I.T. Technics, Groningen, The Netherlands) in the neck flexors, and the neck extensors as previously described (van Leeuwen-Segarceanu et al. 2012). Concomitant diseases such as Parkinson’s disease that have been associated with DHS were excluded by neurological examination. All neurological examinations have been carried out by the same neurologist (P.T.) with long-standing clinical experience who diagnosed radiation-induced DHS based on clinical and neurological features.

### Additional examinations

All exposed CCS were asked to fill out two short questionnaires (Neck Disability Index (NDI) and EORTC FA 12 questionnaire) to assess symptoms of cervical muscle atrophy and to exclude concomitant cancer-related fatigue. For the EORTC FA 12 questionnaire, a score of 12 points corresponds to the least restriction with regard to fatigue symptoms and 48 points to the greatest possible restriction. Additionally, CCS were asked to answer a set of lifestyle questions to evaluate the implementation of the activity recommendations from the American Cancer Society in their daily life (more than 150 min of moderate or 75 min of intense exercise per week) (Rock et al. 2012).

All CCS with abnormal results in the neurological examination were offered an MRI scan for further evaluation. CCS with normal neurological results who received an MRI scan of the head/neck for other reasons (e.g. regular follow-up in brain tumor patients) served as a control group (s. table A.4). All MRI scans were evaluated by neuroradiologists with several years of experience and reassessed without knowledge of the neurological examination by the same neuroradiologist (T.B.) with long-standing expertise.

### Statistical analysis

The programming language R (version 4.2.2) was used for data analysis (R Core Team 2022). For the analysis of continuous variables, the median and the range were considered. Boxplots and scatter plots were created by package “ggplot2” (Wickham 2016). For graphical representation, a fractional polynomial is estimated and mapped for the scatterplots using the package “mfp” (Royston and Altman 2018). In regard to the relationship between radiation dose and neck extension force and between radiation dose and neck flexion force, the model (radiation dose/100)^−2^ was used. For categorical data, the absolute number as well as the percentage was given.

A Mann–Whitney-*U*-test was performed to descriptively examine the difference in neck to thigh circumference and the neck, thigh and waist circumferences in the exposed group compared to the unexposed.

The scores achieved on the NDI questionnaire were converted into percentages by dividing the score achieved by the maximum score. This result was then multiplied by 100 to obtain a percentage score.

In addition, CCS were divided into three groups based on the severity of their disability as measured by the achieved NDI score. For this purpose, the criteria used in Harris et al. (2022) were applied. CCS with scores of 4 or less were assigned to the group with no disability. CCS with scores between 5 and 14 points were considered mildly disabled, while those with scores greater than 14 points were considered moderately disabled.

This study was performed in line with the principles of the Declaration of Helsinki. Approval was granted by the Ethics Committee of the University of Luebeck (reference number 14-180). Informed consent was obtained from all survivors included in the study.

## Results

### Survivors’ characteristics

The study recruited 205 long-term survivors of childhood, adolescent and young adult cancer between May 2020 and April 2022. In this cohort, 26 CCS (12.7%) were 18 years or older at the time of diagnosis.

The group of exposed CCS consisted of 41 CCS (20%) with cervical RT and RT doses of at least 19 Gy. The group of unexposed CCS consisted of 164 CCS (80%), including three CCS (1.5%) who also received neck radiation, but with doses less than 19 Gy. The data set of the exposed CCS included 25 (61.0%) women and 16 (39.0%) men. The unexposed CCS consisted of 107 (65.2%) women and 57 (34.8%) men. A more detailed overview of the characteristics of the exposed and unexposed CCS is shown in Table 2.

**Table 2:** Survivors’ characteristics (*n* = 205)

Characteristics	Total cohort (*n* = 205)	Exposed survivors (*n* = 41)	Unexposed survivors (*n* = 164)
Sex			
Female	132 (64.4%)	25 (61.0%)	107 (65.2%)
Male	73(35.6%)	16 (39.0%)	57 (34.8%)
Median age at cancer diagnosis, years			
Median (range)	10 (0–34)	13 (1–30)	9 (0–34)
Time since diagnosis, years			
Median (range)	16 (0–57)	18 (0–39)	16 (4–57)
Underlying cancer diseases^a^			
Lymphomas	41 (20.0%)	20 (48.78%)	21 (12.8%)
Leukemia	62 (30.24%)	4 (9.76%)	58 (35.37%)
Brain tumors	47 (22.93%)	15 (36.59%)	32 (19.51%)
Sarcomas	32 (15.61%)	2 (4.88%)	30 (18.3%)
Embryonal tumors	13 (6.34%)	0 (0%)	13 (7.9%)
Rare tumors	10 (4.88%)	0 (0%)	10 (6.1%)
Chemotherapy			
Yes	180 (87.8%)	41 (100%)	139 (84.76%)
No	22 (10.73%)	0 (0%)	22 (13.41%)
Unknown	3 (1.46%)	0 (0%)	3 (1.83%)
OP cervical			
Yes	8 (3.9%)	3 (7.32%)	5 (3.05%)
No	194 (94.63%)	38 (92.68%)	156 (95.1%)
Unknown	3 (1.46%)	0 (0%)	3 (1.8%)
Neck Radiotherapy			
Yes (> 19 Gy)	41 (20.0%)	41 (100%)	0 (0%)
Yes (< 19 Gy)	3 (1.46%)	0 (0%)	3 (1.8%)
No	161 (78.54%)	0 (0%)	161 (98.2%)
BMI			
Median (range)	23.975 (14.9–49.2)	26 (16.1–33.2)	23.5 (14.9–49.2)
Neck circumference, cm			
Median (range)	33 (26–55)	32 (26–48)	33 (27–55)
Thigh circumference, cm			
Median (range)	57.5 (37–79)	56 (37–75)	58 (39–79)
Waist circumference, cm			
Median (range)	83 (56.5–135)	86 (66–120)	82.15 (56.5–135)
Ratio neck to thigh circumference			
Median (range)	0.59 (0.43–0.84)	0.57 (0.43–0.78)	0.60 (0.46–0.84)
Ratio neck to waist circumference			
Median (range)	0.41 (0.31–0.57)	0.38 (0.31–0.44)	0.42 (0.31–0.57)

^a^Lymphomas: Hodgkin lymphoma (HL), T-lymphoblastic lymphoma, non HL; Leukemia: acute lymphoblastic leukemia (ALL), acute myeloid leukemia (AML), acute promyelocytic leukemia (APL), bilinear leukemia, chronic myeloid leukemia (CML), B-lymphoblastic leukemia; Brain tumors: Medulloblastoma, intracranial germinoma, astrocytoma, anaplastic ependymoma, atypical teratoid/rhabdoid tumor, craniopharyngioma, glioma, dysembryoplastic neuroectodermal tumor, dysembryoplastic neuroepithelial tumor; Sarcomas: osteosarcoma, rhabdomyosarcoma, clear cell sarcoma, Ewing sarcoma, Embryonal tumors: nephroblastoma, germinoma, germ cell tumor, hepatoblastoma, neuroblastoma; Rare tumors: Langerhans cell histiocytosis, adenocarcinoma, thyroid carcinoma, hepatocellular carcinoma HCC, infantile myofibromatosis, Primitive neuroectodermal tumor (PNET)

### Therapy data

Overall, the median age of patients at first cancer diagnosis was 10 years (13 years exposed, 9 years unexposed). A median of 16 years have passed since diagnosis (18 years exposed, 16 years unexposed).

Of the 41 exposed CCS, almost half were affected by Hodgkin’s lymphoma (20 patients; 48.78%). The remaining CCS were survivors of leukemia (4; 9.76%), brain tumors (15; 36.59%) and sarcomas (2; 4.8%). Detailed data for the exposed and unexposed CCS are given in Table 2.

All 41 (100%) exposed CCS underwent chemotherapy, compared with 139 (84.8%) of the unexposed. The median radiation dose to the neck in the exposed CCS was 30 Gy (19.8–68 Gy). All brain tumor patients included in the exposed cohort have been treated with craniospinal irradiation. Cervical surgery was performed in 3 (7.32%) CCS of the exposed and in 5 (3.05%) of unexposed CCS.

### Physical examinations

**Fig. 3:**
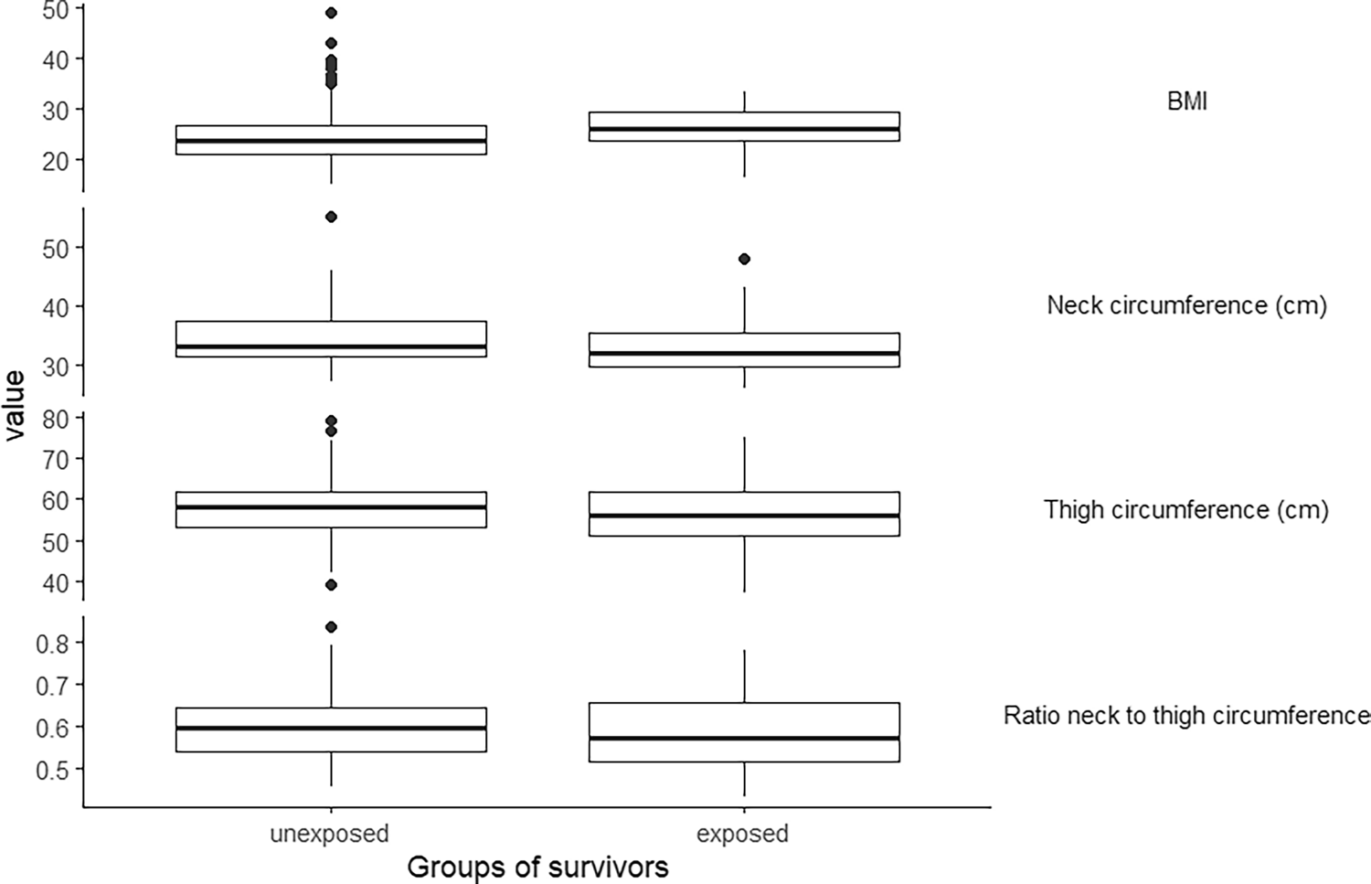
Physical examinations of the exposed (n = 41) and unexposed (n = 164) survivors

The BMI of exposed CCS, with a median of 26 kg/m^2^, was higher than the BMI of unexposed CCS, with 23.5 kg/m^2^. In addition, the waist circumference of exposed CCS, with a median of 86 cm, was higher than that of the unexposed, with 82.15 cm [*p* = 0.087, 95%-KI: (− 1.00, 9.50)]. However, the median thigh circumference of the exposed CCS was 56 cm, which was lower than that of the unexposed, whose median was 58 cm [*p* = 0.462, 95%-KI: (− 4.00, 2.00)]. The neck circumference of the exposed CCS with 32 cm was also lower than the measured median of 33 cm for the unexposed [*p* = 0.013, 95%-KI: (− 3.20, − 0.50)]. When looking at the ratio of the neck and thigh circumferences, it was noticeable that both the median at 0.38 and the maximum of the range with 0.31–0.44 were lower for the exposed CCS than for the unexposed at 0.42 and 0.31–0.57, respectively (Fig. 3). The Mann–Whitney-*U*-test resulted in a descriptive *p*-value of 0.2847 with a 95% confidence interval of [− 0.0508, 0.0170] for the comparison between exposed and unexposed CCS with respect to the ratio of neck to thigh circumference.

The ratio of neck to thigh circumference was shown to be robust and independent of BMI in the exposed CCS (fig. A.1). There was no apparent relationship between the ratio of neck to thigh circumference and radiation dose and neck flexion strength.

**Fig. 4:**
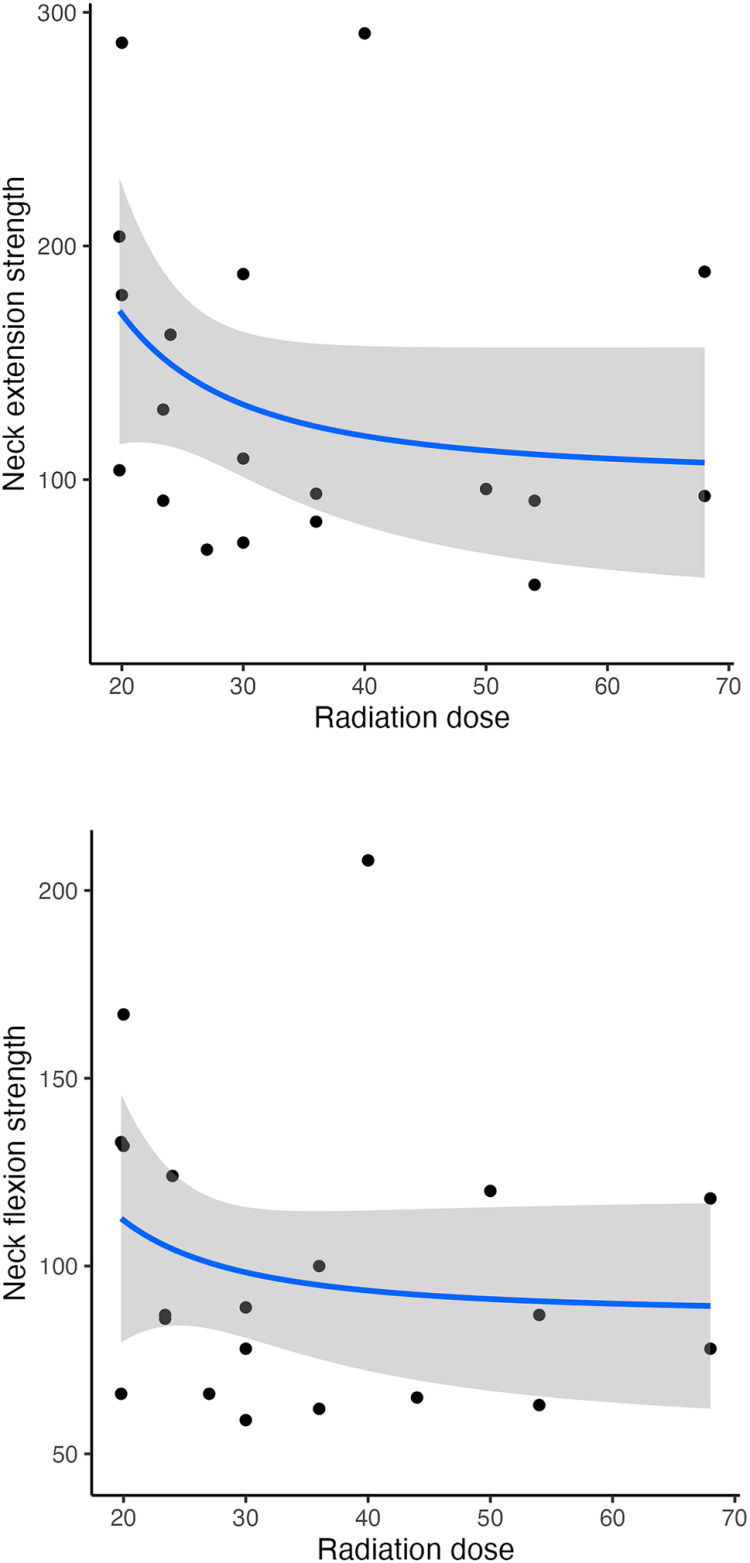
Radiation dose of the exposed survivors (n = 41) compared to the neck flexion and extension strength. Lines were fitted with a fractional polynomial

Supplemental variables were collected for the exposed CCS, which are shown in Table 3. Overall, 16 (39.02%) exposed CCS had known neurological comorbidities and 35 (85.3%) had known endocrinological comorbidities. There were seven (17.07%) CCS who reported a positive family history of neurological diseases. RT doses of about 30 Gy appeared to result in lower neck strength in most exposed CCS when compared to doses lower than 30 Gy (Fig. 4).

**Table 3:** Supplementary examinations of the exposed survivors, divided into normal and abnormal results in the neurological examination

Characteristics	Normal (*n* = 36)	Abnormal (*n* = 5)
Age at diagnosis, years		
Median (range)	13 (1–30)	14 (2–16)
Time since diagnosis, years		
Median (range)	17 (0–39)	30 (7–39)
Radiation dose, Gy		
Median (range)	27 (19.8–68)	30 (19.8–44)
BMI		
Median (range)	26.05 (16.1–33.2)	21.1 (18.5–29.1)
Ratio neck to thigh circumference		
Median (range)	0.58 (0.43–0.78)	0.56 (0.46–0.64)
Neurological comorbidities		
Yes	13 (36.1%)	3 (60%)
No	22 (61.1%)	1 (20%)
Unknown	1 (2.7%)	1 (20%)
Endocrinological comorbidities		
Yes	31 (86.1%)	4 (80%)
Family history		
Positive	5 (13.8%)	2 (40%)
Negative	23 (63.8%)	2 (40%)
Not asked	8 (22.2%)	1 (20%)
NDI		
Filled	29 (80.5%)	4 (80%)
Median (range)	8% (0–32%)	25% (10–32%)
NDI categories		
No disability	15 (41.6%)	0 (0%)
Mild disability	12 (33.3%)	3 (75%)
Moderate disability	2 (5.5%)	1 (25%)
EORTCA FA 12		
Filled	29 (80.5%)	3 (60%)
Median (range)	19.5 (12–32)	26 (23–28)
Neurological examination		
Yes	26 (72.2%)	5 (100%)
No	9 (25.0%)	0 (0%)
Not assessed	1 (2.7%)	0 (0%)
Subjective problems head elevation		
Yes	3 (8.3%)	2 (40%)
No	22 (61.1%)	2 (40%)
Not assessed	11 (30.5%)	1 (20%)
Subjective failures arms motor		
Yes	4 (11.1%)	2 (40%)
No	20 (55.5%)	2 (40%)
Not assessed	12 (33.3%)	1 (20%)
Subjective failures arms sensible		
Yes	4 (11.1%)	2 (40%)
No	20 (55.5%)	2 (40%)
Not assessed	12 (33.3%)	1 (20%)
Sensibility		
Abnormal	3 (8.3%)	1 (20%)
Normal	21 (58.3%)	4 (80%)
Not performed	12 (33.3%)	0 (0%)
Neck flexion strength in N		
Median (range)	87 (57–208)	62 (47–101)
Neck extension strength in N		
Median (range)	106.5 (53–291)	73 (34–100)
MRI executed	7 (18.9%)	5 (100%)

### Questionnaires

The Neck Disability Index (NDI) questionnaire was completed by 33 (80.5%) of the exposed CCS. They scored a median of 12% with a range of 0–40%. When categorizing the scores into none, mild, and moderate disability, 15 (36.5%) CCS were assigned to the no disability group. There were 15 (36.5%) CCS with a mild disability and 3 (7.3%) with a moderate disability.

There was no apparent graphical relationship between the NDI questionnaire score and radiation dose or time elapsed since diagnosis. Furthermore, there appeared to be no relationship between the NDI result and neck extension or flexion strength (fig. A.2).

The cancer-related fatigue questionnaire (EORTC FA 12) for the assessment of quality of life was answered by 31 (75.6%) exposed CCS, who scored a median of 20 points (12–32).

In total, 22 of the exposed CCS (53,7%) answered lifestyle questions to evaluate the implementation of the activity recommendations from the American Cancer Society. Out of this group, 7 (31,8%) met the activity criteria.

### Neurological examinations

In addition, the ability to perform three Hammersmith Functional Motor Scale (HFMS) exercises was examined in the exposed CCS. All three exercises were performed by 28 (68.3%) CCS (table A.1). Exercise 12 [lifts head from prone (arms down by sides)] could not be performed by only one CCS. Exercise 13 (achieves four-point kneeling-head up) could be performed by all CCS. However, exercise 15 (gets to sitting from lying through side lying) could not be performed by 2 CCS, including the CCS who could not perform exercise 12.

Supplemental, this CCS was the only one to exhibit grade 3 on almost all strength measurements in the neck, arm, and hand. The remaining CCS almost exclusively exhibited the highest grade 5 (table A.2).

In the subjective assessment of problems regarding head elevation abilities, five (12.2%) exposed CCS indicated problems. For arm failures, six (14.6%) indicated motor problems and sensory problems. Abnormal sensitivity was also noted in 4 (9.8%) CCS (Table 3).

This also showed the results of the strength measurements performed on 28 (68.3%) CCS. They achieved a median of 86.5 N (47–208 N) for neck flexion and a median of 100 N (34–291 N) for neck extension.

Furthermore, when examining reflexes, 3 CCS (7.3%) showed increased reflexes, and 6 (14.6%) showed decreased reflexes (table A.3).

In summary, 5 CCS (2.4% of all, 12.2% of the exposed CCS) showed abnormal results in the neurological examination consistent with radiation-induced DHS and were referred to an MRI scan. None of the CCS included in the study was diagnosed with Parkinson’s disease.

### MRI examinations

A total of 13 neck MRI examinations were performed (5 recommended by the neurologist, 8 during regular follow-up). In four MRI, symmetric muscle atrophy was present. All of these CCS had abnormal results in the neurological examinations so that four out of five CCS (80%) with neurological abnormalities revealed MRI abnormalities. Only one CCS with abnormalities in the clinical examination showed a regular MRI scan. In four CCS without neurological examination, MRI was performed during regular follow-up and revealed no abnormalities in neck muscles. Three out of six (50%) CCS following head or neck surgery showed muscle asymmetry in the area of the surgical access but, apart from that, no signs of muscle atrophy. A neurological examination could only be performed in one out of these three CCS and revealed normal results (table A.4).

### Characteristics of CCS with abnormal results in the neurological examinations

**Fig. 5:**
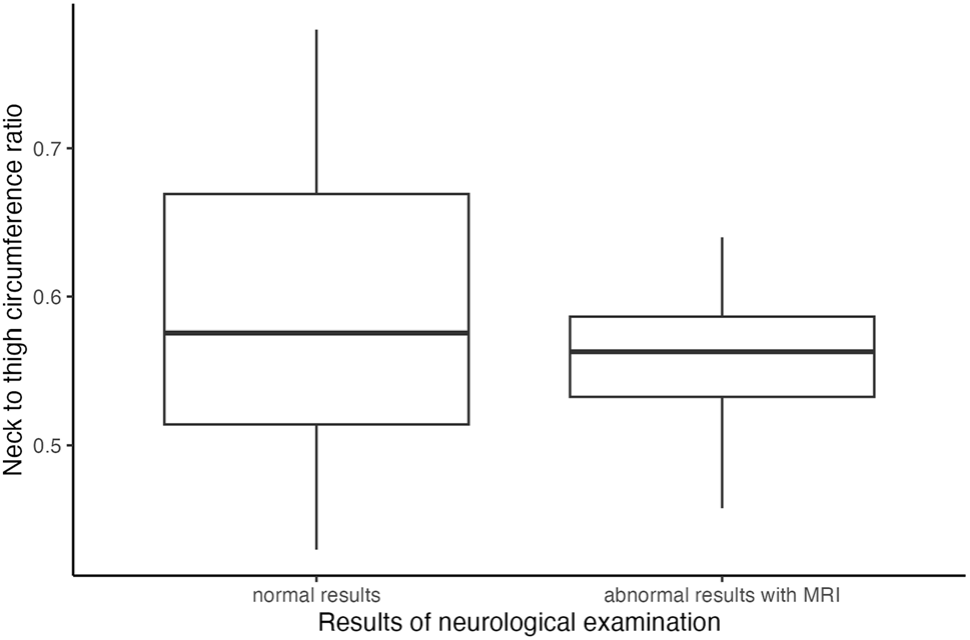
Ratio of neck circumference to thigh circumference in exposed survivors (n = 41) divided into neurological examination results normal (36; 87.8%) and abnormal (5; 12.2%)

The obtained neurological characteristics of the 5 (12.2%) exposed CCS with abnormal results in the neurological examinations compared with the 36 (87.8%) exposed CCS with normal results are shown in Table 3. The median radiation dose of 30 Gy (19.8–44 Gy) of those with abnormal results was higher than the median 27 Gy (19.8–68 Gy) of those with normal results. In addition, the median time since diagnosis was longer in the exposed CCS with abnormal neurological results (30 years, range 7–39 years) than in the CCS with normal results (17 years, range 0–39 years). The median neck-to-thigh circumference ratios of the CCS with abnormal results in the neurological examinations were lower than those of the neurologically normal CCS (Fig. 5).

Of the CCS with abnormal results in the neurological examination, the EORTC questionnaire could be performed in three. These CCS did not achieve remarkably higher values (23, 26 and 28) than the other exposed CCS.

Of the CCS with abnormal results in the neurological examination, 2 (40%) answered the lifestyle questions and one met the criteria. Among the CCS with normal results in the neurological examination, 20 made statements (55.6%), of whom 6 met the activity criteria.

## Discussion

Worldwide, the number of patients surviving childhood cancer has been steadily increasing during the last decades (Siegel et al. 2022). However, long-term health is compromised by chronic health conditions arising from cancer treatment years to decades after the cessation of therapy, resulting in a reduced quality of life as well as higher morbidity and mortality in CCS (Bhakta et al. 2017). Risk-adapted surveillance programs are intended to prevent (or at least facilitate early diagnosis of) possible late effects and are tailored specifically to patients’ treatment exposure and risk factors (Gebauer et al. 2020). They are based on large analyses of CCS’ health status in adulthood but often struggle with a retrospective and/or questionnaire-based approach and with the lack of an adequate control/reference group. Furthermore, these analyses are challenged by the fact that childhood cancer is generally considered to be a rare condition whereas many chronic health conditions (and possible late effects) such as diabetes mellitus occur regularly in the general population (although more often later in life) so that demonstration of a true rise in the incidence of these conditions requires large numbers of patients (Landier et al. 2018). As a consequence, rare late effects, especially those manifesting decades after treatment exposure, are difficult to detect and, due to a lack of awareness, may even escape correct diagnosis.

DHS represents a rare condition in the context of long-term survival after exposure to neck radiotherapy. Its main characteristics are weakness and atrophy of cervical muscles resulting in an inability to extend the neck so that the head flexes forward (Grimm and Chamberlain 2011). The severe atrophy varies from patient to patient and likely results from a combination of damage to the motor nerve and/or the muscle itself (Stubblefield 2011). It has predominantly been documented in HL survivors (mainly due to their good cancer-related prognosis) but is primarily associated with high-dose RT and a long interval between treatment exposure and first symptoms (van Leeuwen-Segarceanu et al. 2012; Furby et al. 2010). In line with this, we also observed in our cohort that CCS with radiation-induced DHS had been exposed to a higher median radiation dose and the median time since diagnosis was longer in this group. Although younger age at radiation exposure has been identified as a risk factor for several late effects, it has not been linked to radiation-induced DHS yet (Krasin et al. 2010; Steinmeier et al. 2019). Head and neck cancer survivors may also be affected by DHS although their neck extensor weakness appeared to be less severe, presumably because of the exclusion of thoracic paraspinal muscles, which are important in neck extension, from the radiation field (Stubblefield 2011). Consequently, in this study, we also included brain tumor survivors treated with high doses of craniospinal RT; however, only one of those CCS was affected by radiation-induced DHS and showed signs of muscle atrophy in the MRI.

Of note, late neurologic complications of RT exposure are not limited to DHS but may cause muscle and/or nerve damage within the entire radiation field. This is underlined by the fact that one HL survivor in our study who has been treated with mantle field RT was diagnosed with late radiation myelopathy during a neurological work-up resulting in progressive paralysis of the right arm and leg and one other HL survivor was diagnosed with brachial plexopathy during the DHS work-up, constituting another rare complication of HL treatment that has been described as an acute as well as a late complication of RT (Grimm and Chamberlain 2011).

All exposed CCS received chemotherapy treatment during their cancer therapy. As most of these CCS were lymphoma, brain tumor and sarcoma survivors, they were exposed to agents with neurotoxic potential such as vinca alkaloids or platinum compounds that were, in the case of medulloblastoma and sarcoma patients, administered concurrently with RT. However, almost all CCS in our cohort affected by radiation-induced DHS were lymphoma survivors (4; 80%) who received RT after the end of chemotherapy treatment.

Vinca alkaloids or platinum compounds are mainly associated with acute neurotoxicity often manifesting as peripheral neuropathy (Grimm and Chamberlain 2011). Vincristine, for example, predominantly affects longer neurons in the (lower) limbs. In many cases, symptoms develop already after a few administrations and often disappear again after the end of therapy although some CCS are affected by long-term neurologic symptoms (Velde et al. 2017). To date, based on the literature available, radiation-induced DHS has not been linked to chemotherapy (Grimm and Chamberlain 2011). However, as radiation-induced DHS is mainly described in patients with HL who were treated with mantle irradiation and chemotherapy as well, a synergistic effect of both treatment modalities has been discussed before, mainly in patients with early onset DHS (Hashimoto et al. 2012). Further analyses comparing cancer survivors who were treated only with radiotherapy and CCS who received both therapies could reveal a potential deteriorating effect of concomitant chemotherapy.

Newer treatment protocols for childhood cancer have reduced or even eliminated radiotherapy wherever possible without risking an uncontrollable rise in relapses to prevent RT’s effect on exposed tissue which may result in subsequent malignancies occurring in the former radiation field as well as in organ dysfunction as, for example, hypothyroidism or hypopituitarism (Gebauer et al. 2019; Stone et al. 2003; Schellong et al. 1999).

Following the implementation of combination chemotherapy protocols for HL, “extended” field RT with doses of 36–40 Gy was replaced by lower dose (21–25 Gy) involved field RT to reduce (musculoskeletal) toxicities (Jairam et al. 2013). During the last two decades, conventional RT has been increasingly replaced by modern precision techniques including intensity-modulated radiotherapy (IMRT) and volumetric modulated arc therapy (VMAT), which are now the current treatment standard in most radiation oncology centers (Steinmeier et al. 2019). Moreover, several imaging modalities including PET-CT are used to further increase the precision of treatment planning and delivery (image-guided radiotherapy; IGRT). These advances in RT techniques can lead to a significant reduction of the radiation doses to normal tissues and organs at risk and, consequently, to a reduction of the risk of late radiation-related toxicities (Steinmeier et al. 2019). However, since precision RT techniques were not available in many centers about 20 years ago, it will still take some more time to properly identify a possible risk reduction for late effects in these younger survivors (Constine et al. 2019). Until then, one has to be aware that many CCS worldwide who received conventional RT to the neck are still alive and at risk of experiencing treatment-related DHS.

While analyzing data from CCS treated before the computed tomography treatment planning era, it is important to consider that it was common for reported radiation doses to be those prescribed to the tumor, rather than doses to adjacent critical normal tissues as pointed out by PENTEC (Pediatric Normal Tissue Effects in the Clinic), an international collaboration analyzing normal tissue radiation dose-volume response relationships for childhood cancer patients (Constine et al. 2019). Also, correction for tissue homogeneity was not routinely carried out at that time and uncertainties resulting from a lack of three-dimensional planning occurred so that previously reported doses might be an under- or overestimating of the applied radiation dose (Constine et al. 2019).

Based on the literature available, which consists only of small cases studies, analysis in small preselected cohorts or case reports, the incidence of radiation-induced DHS in long-term CCS appears to be low. However, it remains unclear whether this is due to the underdiagnosis of this disease (due to a lack of awareness and specific recommendations) or whether it reflects a truly low incidence. Furthermore, no diagnostic algorithm for this condition is established yet so that affected patients often have to undergo various diagnostic modalities including invasive procedures such as muscle biopsy, to exclude other conditions associated with DHS, such as Parkinson’s disease (see Table 1).

In this study, we prospectively assessed and examined almost all CCS regularly visiting our LTFU clinic between May 2020 and April 2022 and evaluated a diagnostic algorithm for all CCS after neck radiotherapy. The diagnostic algorithm was developed based on the diagnostic tools used in former studies on radiation-induced DHS (see Table 1 and Fig. 2). EMG and muscle biopsy have not been regularly included in the diagnostic algorithm as former studies revealed conflicting results for EMG alterations and primarily non-specific abnormalities in histopathological studies as outlined in the “Methods” sections of this paper. However, histologic evaluation has been considered as “ultima ratio” for unsolved cases, which, in the absence of any further clinical, genetical or neurological hint for an alternative disorder, has not been performed in this study. We also included measurement of neck circumference that, in relation to the nonirradiated thigh, turned out to be an easy tool to clinically assess muscle atrophy in formerly irradiated CCS. Neck circumference has been proposed as a screening tool for central obesity as it strongly correlated with waist circumference in former studies (Anothaisintawee et al. 2019). In our study, although exposed CCS were more obese, neck circumference as well as neck/thigh ratio were lower than in unexposed survivors. However, as no clear difference between these two groups could be observed, the role of these measurements for early diagnosis of radiation-induced DHS has to be verified in larger cohorts in future studies.

Analyzing the Neck Disability Index (NDI) scores, less than half of the patients (37%) showed no impairment, which corresponds well to former studies revealing similar values (44% with no impairment) in head and neck cancer survivors (Harris et al. 2022). In other words, more than 50% of CCS exposed to neck RT indicated neck impairment and pain emphasizing the need for better awareness and management of neck disability even if no apparent radiation-induced DHS is diagnosed (yet). Pain associated with neck extensor weakness is assumed to be multifactorial due to fatigue and painful spasm of the muscles as well as functional overload and muscle imbalance (Stubblefield 2011). Consequently, the mainstay of neck extensor weakness treatment is physical therapy to emphasize postural retraining although, as the fibrotic process cannot be directly affected, weakness and dysfunction often progress over time so that ultimately, some patients need a cervical orthotic to maintain the head in an upright position (Stubblefield 2011; Kim and Stubblefield 2019). In our cohort, no patient showed severe weakness requiring a prescription for a headmaster cervical collar although several survivors reported worsening of pain and muscle weakness as the day progressed which has been described as a typical feature of radiation-induced DHS (Stubblefield 2011). Timely initiation of adequate treatment might delay or even prevent severe neck impairment emphasizing the importance of an early diagnosis.

Our study confirmed, for the first time, the presumed low incidence of radiation-induced DHS in an unselected cohort of CCS with only 2.4%. However, in the subgroup of patients receiving neck RT with at least 19 Gy, about 12% presented with clinical and neurological symptoms consistent with radiation-induced DHS. MRI confirmed signs of cervical muscle atrophy in 80% of these cases, whereas muscle atrophy could not be detected in CCS without clinical or neurological signs of DHS who received MRI as part of their routine cancer follow-up. Few former studies indicated a much higher prevalence of radiation-induced DHS in HL survivors up to 83% in a recent analysis in which, however, diagnosis of DHS was solely based on subjective weakness in head elevation or weak cervical paraspinal muscle contraction demonstrated during palpation on physical exam (Enam et al. 2022). Furthermore, only survivors visiting a cancer rehabilitation outpatient clinic for functional impairments have been included (Enam et al. 2022) so that, similar to another study on progressive muscle atrophy in HL survivors in which a relevant share of potential survivors could not be included in the study due to multiple reasons (van Leeuwen-Segarceanu et al. 2012), data did not allow to draw conclusions on the true incidence of radiation-induced DHS in an unselected cohort of CCS. Our proposed algorithm for radiation-induced DHS diagnosis showed good reliability with a high concordance between clinical/neurological and MRI results as well as practicability in a clinical setting without risks of radiation exposure or invasive procedure. A focused anamnesis and clinical and neurological examination were sufficient to diagnose radiation-induced DHS and the MRI scan did not reveal additional information although this approach needs to be further evaluated in larger cohorts. Furthermore, a lower neck circumference was associated with clinical and neurological signs of neck muscle atrophy and could serve as an easy method to screen for neck muscle atrophy in survivors exposed to neck RT that should be verified in larger studies in the future.

When interpreting the results of our study, its limitations should be considered. The performance of the study during the COVID-19 pandemic resulted in major restrictions affecting work in our outpatient clinic. As one consequence of limited time during the clinic stay, neurological assessment as well as completing the questionnaires could not be performed in all patients with neck RT exposure.

Furthermore, the systematic literature search was challenged by an inconsistent nomenclature (“radiation induced myopathy”; Ghosh and Milone 2015).

In summary, this study presents the first study prospectively assessing the incidence of radiation-induced DHS in an unselected as well as in a RT exposed cohort of long-term CCS. A relevant proportion of long-term CCS formerly treated with neck RT is affected by this sequela although it remains a rare condition in the overall group of CCS. A diagnostic algorithm including a clinical and neurological assessment facilitates diagnosis and, if integrated into standard surveillance programs, initiation of early treatment of radiation-induced DHS to optimize risk-adapted and comprehensive long-term follow-up care of CCS.

### Supplementary Information

Below is the link to the electronic supplementary material.

Supplementary file1 (JPG 130 KB)

Supplementary file2 (JPG 95 KB)

Supplementary file3 (DOCX 21 KB)

Supplementary file4 (DOCX 24 KB)

Supplementary file5 (DOCX 21 KB)

Supplementary file6 (DOCX 24 KB)

## Data Availability

The anonymised data that support the findings of the study are available from the corresponding author upon reasonable request.
