# Supplementary figures and images for: Proposal of a diagnostic algorithm for radiation-induced dropped head syndrome in long-term childhood cancer survivors based on a prospective study in a specialized clinical setting and a review of the literature
Source: J Cancer Res Clin Oncol. 2023 Nov 10;149(20):17865–79. doi: 10.1007/s00432-023-05480-w (PMC10725355; doi:10.1007/s00432-023-05480-w)

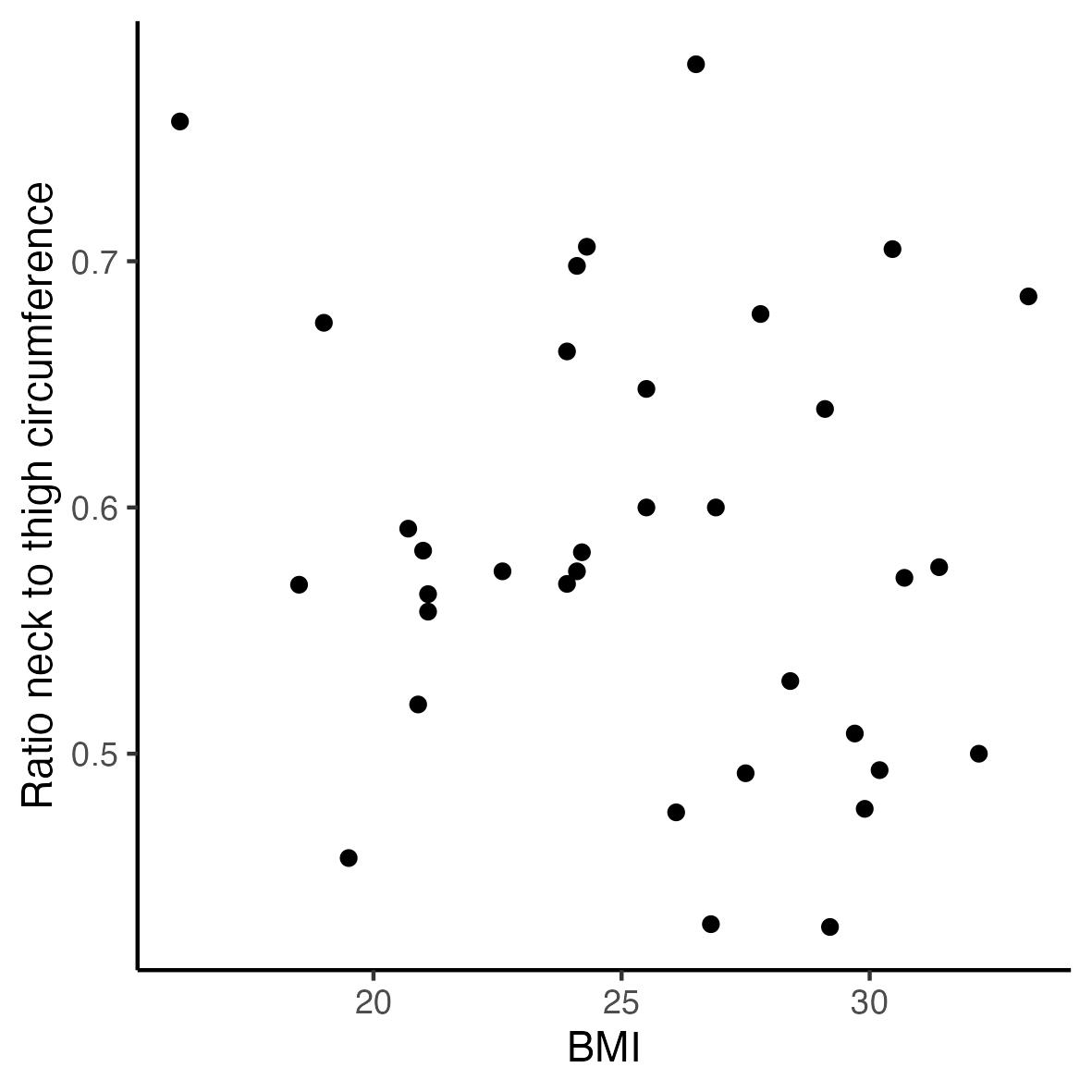

Supplement: Supplementary file 1 — Supplementary file1 (JPG 130 KB) [file 432_2023_5480_MOESM1_ESM.jpg]

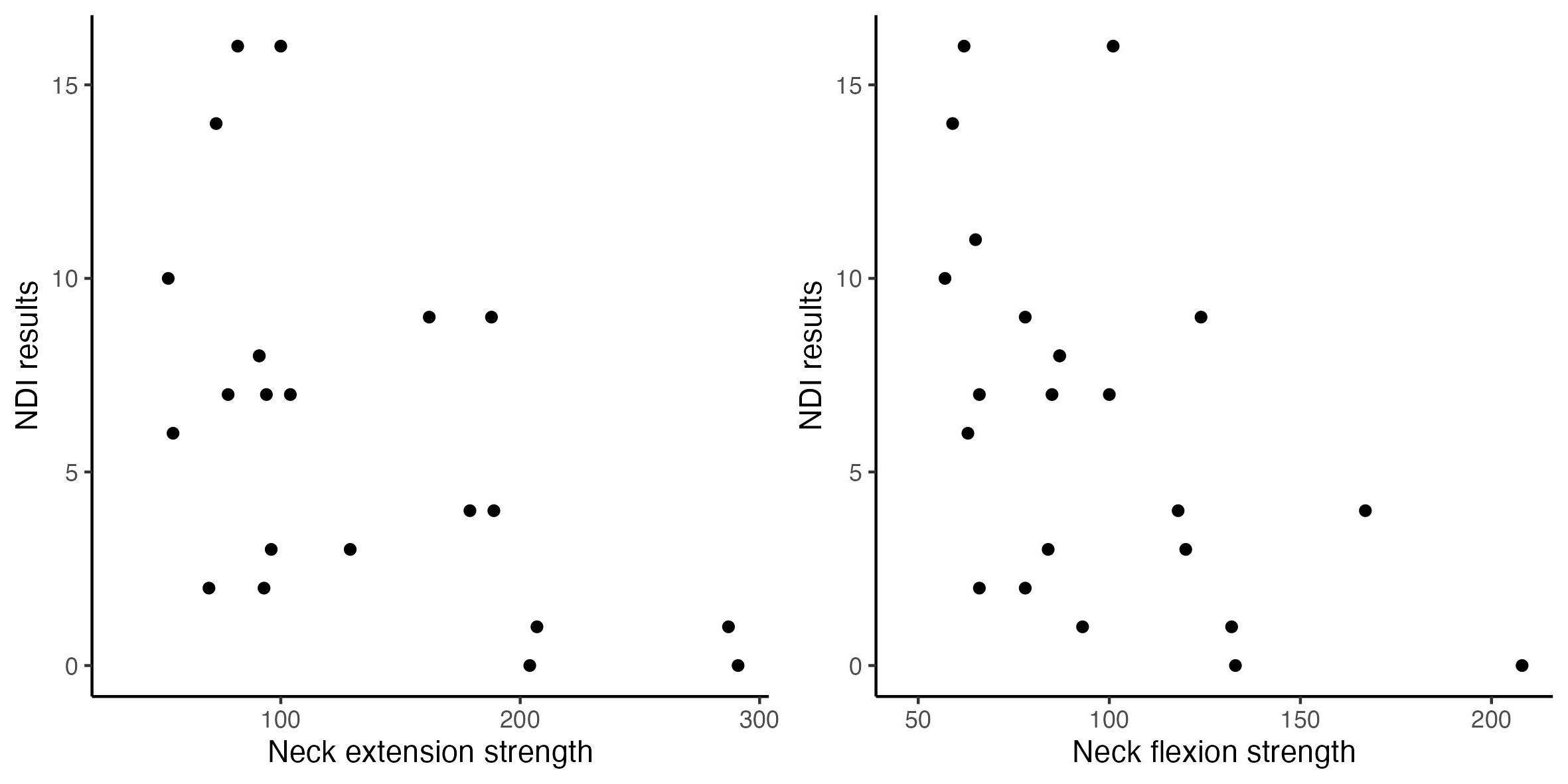

Supplement: Supplementary file 2 — Supplementary file2 (JPG 95 KB) [file 432_2023_5480_MOESM2_ESM.jpg]
