# Supplementary material for: Proposal of a diagnostic algorithm for radiation-induced dropped head syndrome in long-term childhood cancer survivors based on a prospective study in a specialized clinical setting and a review of the literature
Source: J Cancer Res Clin Oncol. 2023 Nov 10;149(20):17865–79. doi: 10.1007/s00432-023-05480-w (PMC10725355; doi:10.1007/s00432-023-05480-w)
Supplement: Supplementary file 3 — Supplementary file3 (DOCX 21 KB) [file 432_2023_5480_MOESM3_ESM.docx]

**Table A.1.** HFMS in survivors (n=41)

| Exercises | 0 | 1 | 2 | Unknown |
| --- | --- | --- | --- | --- |
| Item 12 | 2.4% | 0% | 65.8% | 31.7% |
| Item 13 | 0% | 0% | 68.3% | 31.7% |
| Item 15 | 4.8% | 0% | 63.4% | 31.7% |
| Item 12: Lifts head from prone (arms down by sides); Item 13: Achieves four-point kneeling-head up; Item 15: Gets to sitting from lying through side lying | | | | |
