# Supplementary material for: Proposal of a diagnostic algorithm for radiation-induced dropped head syndrome in long-term childhood cancer survivors based on a prospective study in a specialized clinical setting and a review of the literature
Source: J Cancer Res Clin Oncol. 2023 Nov 10;149(20):17865–79. doi: 10.1007/s00432-023-05480-w (PMC10725355; doi:10.1007/s00432-023-05480-w)
Supplement: Supplementary file 4 — Supplementary file4 (DOCX 24 KB) [file 432_2023_5480_MOESM4_ESM.docx]

**Table A.2.** Strength measurements in survivors (n=41)

| Measurements | Grade 1 | Grade 2 | Grade 3 | Grade 4 | Grade 5 | Not possible | Unknown |
| --- | --- | --- | --- | --- | --- | --- | --- |
| Neck extension | 0 | 0 | 0 | 0 | 29 | 1 | 11 |
| Neck bending | 0 | 0 | 0 | 1 | 29 | 0 | 11 |
| Head rotation right | 0 | 0 | 1 | 1 | 29 | 0 | 10 |
| Head rotation left | 0 | 0 | 1 | 1 | 29 | 0 | 10 |
| Shoulder abduction right | 0 | 0 | 1 | 1 | 29 | 0 | 10 |
| Shoulder abduction left | 0 | 0 | 1 | 0 | 30 | 0 | 10 |
| Elbow flexion right | 0 | 0 | 1 | 3 | 27 | 0 | 10 |
| Elbow flexion left | 0 | 0 | 1 | 1 | 29 | 0 | 10 |
| Elbow extension right | 0 | 0 | 1 | 0 | 30 | 0 | 10 |
| Elbow extension left | 0 | 0 | 1 | 0 | 30 | 0 | 10 |
| Wrist flexion right | 0 | 0 | 1 | 1 | 29 | 0 | 10 |
| Wrist flexion left | 0 | 0 | 1 | 0 | 30 | 0 | 10 |
| Wrist extension right | 0 | 0 | 1 | 0 | 30 | 0 | 10 |
| Wrist extension left | 0 | 0 | 1 | 0 | 30 | 0 | 10 |
| Finger spread right | 0 | 0 | 1 | 1 | 29 | 0 | 10 |
| Finger spread left | 0 | 0 | 1 | 0 | 30 | 0 | 10 |
