# Supplementary material for: Proposal of a diagnostic algorithm for radiation-induced dropped head syndrome in long-term childhood cancer survivors based on a prospective study in a specialized clinical setting and a review of the literature
Source: J Cancer Res Clin Oncol. 2023 Nov 10;149(20):17865–79. doi: 10.1007/s00432-023-05480-w (PMC10725355; doi:10.1007/s00432-023-05480-w)
Supplement: Supplementary file 5 — Supplementary file5 (DOCX 21 KB) [file 432_2023_5480_MOESM5_ESM.docx]

**Table A.3.** Examination of the reflexes of the survivors (n=41)

| Examinations | Reduced | Normal | Overactive | Unknown |
| --- | --- | --- | --- | --- |
| Biceps reflex right | 6 | 18 | 3 | 14 |
| Biceps reflex left | 5 | 19 | 3 | 14 |
| Radioperiosteal reflex right | 5 | 19 | 3 | 14 |
| Radioperiosteal reflex left | 5 | 19 | 3 | 14 |
| Triceps reflex right | 4 | 22 | 2 | 13 |
| Triceps reflex left | 4 | 21 | 3 | 13 |
