# Supplementary material for: Proposal of a diagnostic algorithm for radiation-induced dropped head syndrome in long-term childhood cancer survivors based on a prospective study in a specialized clinical setting and a review of the literature
Source: J Cancer Res Clin Oncol. 2023 Nov 10;149(20):17865–79. doi: 10.1007/s00432-023-05480-w (PMC10725355; doi:10.1007/s00432-023-05480-w)
Supplement: Supplementary file 6 — Supplementary file6 (DOCX 24 KB) [file 432_2023_5480_MOESM6_ESM.docx]

| Surgery access visible | Symmetry | Asymmetry affects the following muscles | Symmetric atrophy in the following muscles | Pathological contrast agent uptake in muscles | Muscle edema | Correct patient positioning | Neurological examination |
| --- | --- | --- | --- | --- | --- | --- | --- |
| Yes | No | M. splenius capitis left; M. semispinalis capitis left; both with slight atrophy | No | No | No | Yes | Not assessed |
| Yes | Yes |  | No | No | No | Yes | Not assessed |
| Yes | No | M. rectus capitis posterior minor et mayor; M. obliquus inferior | No | No | No | Yes | Not assessed |
| Yes | Yes |  | deep neck muscles (M. obliquus capitis superior et inferior; Musculus rectus capitis minor et major) | No | No | Head to the left | Abnormal results |
| Yes | No | M. rectus capitis posterior minor et mayor; M. obliquus inferio | No | No | No | Yes | Normal results |
| No | Yes |  | No | No | No | Yes | Abnormal results |
| No | Yes |  | hypotrophic neck muscles | No | No | Yes | Abnormal results |
| No | Yes |  | Autochthonous back muscles on both sides; cervicothoracic with vacate fat formation; very weak neck muscles | No | No | Yes | Abnormal results |
| No | Yes |  | No | No | No | Yes | Normal results |
| No | Yes |  | No | No | No | Yes | Not assessed |
| No | Yes |  | No | No | No | Yes | Normal results |
| No | Yes |  | Autochthonous back muscles on both sides; cervicothoracic with vacate fat formation | No | No | Yes | Abnormal results |
| Yes | Yes |  | No | No | No | Yes | Normal results |

**Table A.4.** MRI results of the CCS (5 recommended by the neurologist, 8 during regular follow-up).
